# Efficacy and safety of resina draconis as an adjuvant therapy for diabetic foot ulcer: a meta-analysis of randomized controlled trials

**DOI:** 10.3389/fphar.2026.1823436

**Published:** 2026-07-22

**Authors:** Shiwei Zhou, Yi Guan, Qiping Yu, Jiaxin Li, Lan Ma, Chengliang Zhu

**Affiliations:** 1 Department of Emergency Medicine, Shaoxing People’s Hospital, Shaoxing, China; 2 School of Medicine, Shaoxing University, Shaoxing, China; 3 College of Clinical Medicine, Affiliated Hospital of Jining Medical University, Jining Medical University, Jining, China; 4 Xinjiang Institute of Technology, Aksu, China

**Keywords:** diabetic foot ulcer, meta-analysis, resina draconis, systematic review, wound healing

## Abstract

**Background:**

Resina Draconis, a traditional Chinese herbal medicine with wound‐healing properties, has been increasingly used as an adjunctive treatment for diabetic foot ulcers (DFUs). This systematic review and meta‐analysis aimed to evaluate the efficacy and safety of Resina Draconis in the treatment of DFUs.

**Methods:**

Five electronic databases were systematically searched for randomized controlled trials (RCTs) published up to December 2025. Eligible studies compared Resina Draconis combined with conventional therapy versus conventional therapy alone in patients with DFUs. Dichotomous outcomes were expressed as risk ratios (RRs), and continuous outcomes as mean differences (MDs), both with 95% confidence intervals (CIs).

**Results:**

Fourteen RCTs involving 1,011 participants were included. The meta‐analysis showed that adjunctive treatment with Resina Draconis significantly improved the overall healing response (RR = 0.28; 95% CI, 0.20–0.39; P < 0.00001), shortened wound‐healing time (MD = −11.66; 95% CI, −21.62 to −1.69; P = 0.02), and reduced serum C‐reactive protein levels (MD = −1.99; 95% CI, −2.99 to −0.99; P < 0.0001). No serious adverse events were reported.

**Conclusions:**

Resina Draconis may be an effective and safe adjunctive therapy for DFUs, potentially enhancing wound healing and reducing systemic inflammation. However, the findings should be interpreted cautiously because of the limited sample sizes and potential publication bias of the included studies. Further large‐scale, rigorously designed RCTs are needed to confirm these results.

**Systematic Review Registration:**

Identifier CRD42020189060.

## 1 Introduction

At present, diabetes mellitus (DM) has become one of the most serious public health crises in the world due to its rapidly increasing prevalence and heavy economic burden. By 2024, the number of DM patients aged 20–79 worldwide has reached 589 million, of which more than 3.4 million patients have died (Duncan et al., 2025). Diabetic foot disease (DFD) is one of the most serious complications of DM. About 20 million DM patients worldwide are complicated with DFD (Lazzarini et al., 2023). The occurrence of DFD greatly increases the disease burden of DM patients, which is mainly manifested in the high disability rate and mortality rate of these patients (Lazzarini et al., 2023; Lazzarini et al., 2024). Diabetic foot ulcer (DFU) is a more serious progressive form of DFD. As one of the most serious consequences of DFU, amputation has an incidence of up to 20% in patients with moderate or severe DFU (Akkus and Sert, 2022). Once the patient is amputated, the 5-year mortality and complication rate are as high as 70% or more (Yu et al., 2023).

DFU is the result of a vicious cycle of nerve ischemic injury and infection based on the pathology of DM. Its core mechanism can be attributed to the triad of diabetic neuropathy, peripheral arterial disease (PAD), and infection (Bandyk, 2018). As the main driving factor of DFU, neuropathy is easy to cause peripheral nerve metabolism and microvascular damage after interacting with the hyperglycemia environment of DM patients, thus progressing into a mixed lesion of sensory, motor and autonomic nerves, resulting in multiple pathological effects (Akkus and Sert, 2022). The high blood glucose level and related abnormal glucose metabolism in DM patients often promote the occurrence and development of DFU through endothelial dysfunction, endothelial-mesenchymal transition, oxidative stress-inflammation axis, epigenetic disorders and other mechanisms (Khan and Jandeleit-Dahm, 2025). It is worth noting that the lower extremity lesions in DM patients are mainly distributed in the lower leg arteries such as the posterior tibial artery and the anterior tibial artery. In the case of the progression of diffuse tibial artery occlusion or the aggravation of proximal artery occlusion, when the perfusion of foot tissue falls to the critical threshold that cannot maintain skin integrity, it will lead to the occurrence of ischemic ulcers or gangrene (Bandyk, 2018).

Clinically, timely and accurate diagnosis and treatment of DFU is an important means to prevent the progression of DFU. The core of the diagnosis of DFU is to have a history of DM combined with foot ulcers, especially those with poor blood glucose control, such as foot skin ulceration involving deep structures such as soft tissue, tendons, and bones. The occurrence of DFU should be highly suspected. At present, the treatment of DFU is diverse. As a basic measure of DFU standard treatment, stabilizing blood glucose level can not only delay the occurrence and development of DFU, prevent the occurrence of complications, but also directly promote the healing of ulcers (Jiang et al., 2023). For patients with severe disease and necrotic tissue, it is recommended to perform surgical debridement and drainage in time to remove necrotic tissue, promote wound healing and prevent further spread of infection (Jiang et al., 2023).

Resina Draconis (RD, also referred to as “Longxuejie” in the Chinese literature) is not a pharmacognostically uniform medicinal material, but a traditional name applied to red resinous products from multiple botanical sources (Sun et al., 2021). RD has been included in the 2020 edition of the Chinese Pharmacopoeia. Published phytochemical studies of Dracaena-derived RD have identified more than 300 metabolites, mainly flavonoids, phenolics, and steroids, including representative metabolites such as loureirin A, loureirin B, loureirin C, loureirin D and cochinchinenin A (Wang et al., 2025). Among them, Loureirin B is considered to be one of the main bioactive components of RD. It has a variety of pharmacological activities, including anti-inflammatory, antioxidant and metabolic regulation, and can potentially improve insulin resistance and promote diabetic wound healing. Because of its important role in regulating immunity, protecting nerves, antibacterial, anti-inflammatory, and promoting wound healing (Sun et al., 2021; Wang et al., 2025; Jin et al., 2025; Guo et al., 2024; Sun et al., 2019), it has been used in the treatment of skin ulcers, atherosclerosis and other diseases in recent years (Wang et al., 2025; Guo et al., 2024). Due to the above effects of RD, there have been studies trying to explore the actual role of RD in the treatment of DFU through randomized controlled trial (RCT). Existing RCTs generally believe that RD plays an important role in promoting skin repair, shortening wound healing time, and anti-inflammation in the treatment of DFU. Feng and his colleagues conducted a meta-analysis (Feng et al., 2023), and the results showed that RD is a safe and effective solution for the treatment of DFU, and the results showed that RD treatment was a safe and effective solution for DFU. Combined with conventional treatment, it can improve the healing rate of DFU, shorten the healing time, and inhibit its further development. However, due to the limited quality and quantity of included studies, higher quality meta-analysis is needed to support this view. Therefore, we will conduct a meta-analysis of more RCTs to guide clinical practice and provide new possibilities for solving the huge disease burden caused by DFU.

## 2 Methods

This systematic review was conducted according to the Preferred reporting Items of Systematic Reviews and Meta-Analysis (PRISMA) guidelines and has been registered on the PROSPERO (Number: CRD42020189060).

### 2.1 Taxonomic and pharmacognostic clarification

According to the Chinese Pharmacopoeia (2020 edition), Xuejie (Draconis Sanguis) is defined as the processed resin obtained from the fruits of Daemonorops draco Blume [Arecaceae].

According to Kew Plants of the World Online (POWO), taxonomic treatments may relate Daemonorops draco Blume to Calamus draco Willd [Arecaceae]. Historical use of the term “dragon’s blood” may also refer to resinous products obtained from other taxa, including Dracaena species and Pterocarpus officinalis Jacq [Fabaceae] (Heinrich et al., 2022).

However, among the 14 RCTs included in this review, species-level identification was rarely reported. Most studies referred only to “Resina Draconis”, “Xuejie”, or “Longxuejie” without providing botanical authentication, pharmacognostic characterization, processing procedures, or phytochemical quality-control information.

Therefore, the intervention evaluated in this review should be interpreted as products labelled as “Resina Draconis”, “Xuejie”, or “Longxuejie” in the original trial reports rather than a single taxonomically validated botanical drug.

### 2.2 Search strategy

We performed a comprehensive search for potentially relevant studies up to December 2025, using PubMed, EMBASE, the Cochrane Library, CNKI and Chinese Biomedical Literature Database (CBM). The following terms were searched (‘draconis resina’ OR ‘Resina Draconis’ OR ‘longxuejie’) AND (‘diabetic foot ulcer’ OR ‘diabetic ulcer’ OR ‘diabetic foot’ OR ‘diabetic foot disease’ OR ‘DFU’ OR ‘chronic ulcer’ OR ‘foot ulcer’ OR ‘wound’). No limitations on language or date were applied. Furthermore, we also found more studies that were relevant from the reference lists of all included articles.

In addition, in order to ensure the high quality of retrieval, we choose to use artificial secondary screening. Two reviewers (S Zhou and Y Guan) independently conducted a second screening, and the selected RCTs were formally included. If there is ambiguity between two RCTs, it is up to a third reviewer (Q Yu) to decide whether the RCT meets the criteria.

### 2.3 Study selection

We set the following inclusion criteria according to the PICOS guidance: (1) The study design is randomized controlled trial (RCT); (2) patients with diabetic foot ulcers of any age; (3) patients in the treatment group were treated with RD on the basis of the control group; (4) the available outcome measures on clinical efficacy. Effective treatment of the disease is defined as the improvement of symptoms or laboratory tests or the clinical cure of the disease.

Studies were excluded when meeting one of the following criteria: (1) animal trial; (2) no available control group; (3) systematic reviews, retrospective studies, comments, or case reports; (4) lack of sufficient data; (5) research with potential conflicts of interest.

### 2.4 Data extraction

The applicable data were abstracted from included studies by two independent authors (S Zhou, Y Guan). The details were as follows: (1) the last name of the first author and publication year; (2) characteristics of participants (age, gender); (3) the grade of diabetic foot ulcers; (4) study methods (interventions in treatment and control groups, frequency and duration); (5) the outcome measures. Only the data for the last time point were extracted when the values were displayed in different time points.

### 2.5 Quality assessment

The risk of bias in trials was referred to the Cochrane Handbook for Systematic Reviews of Interventions (Higgins et al., 2011). Risk of bias of the items for included studies was assessed (A: random sequence generation; B: allocation concealment; C: blinding of patients and personnel; D: blinding of the outcome assessment; E: incomplete outcome data; F: selective reporting). The author carefully assessed the risks of various types of bias and chose one of the three options. The assessment rules are as follows: high risk (the risk may or will affect the accuracy of subsequent data analysis), unclear risk (for various reasons, we cannot objectively or correctly assess the risk of bias, or the risk level of bias is between high risk and low risk), and low risk (the bias does not affect the accuracy of subsequent data processing or is unlikely to affect it).

### 2.6 Statistical analysis

Outcomes were calculated as risk ratio (RR) and mean differences (MD), both with 95% confidence intervals (CI). The range of the heterogeneity index (I^2^) was set to 0%–100%. When I^2^ > 50, statistical heterogeneity was identified. When I^2^ < 50, we used the fixed effect model (FE); otherwise, we used a random effect model (RE) to reduce unreliable outcomes due to high heterogeneity (Zheng et al., 2012). Statistics were considered significant at *P* < 0.05. All data was analyzed with the Review Manager 5.3.

## 3 Results

### 3.1 Database search

**FIGURE 1:**
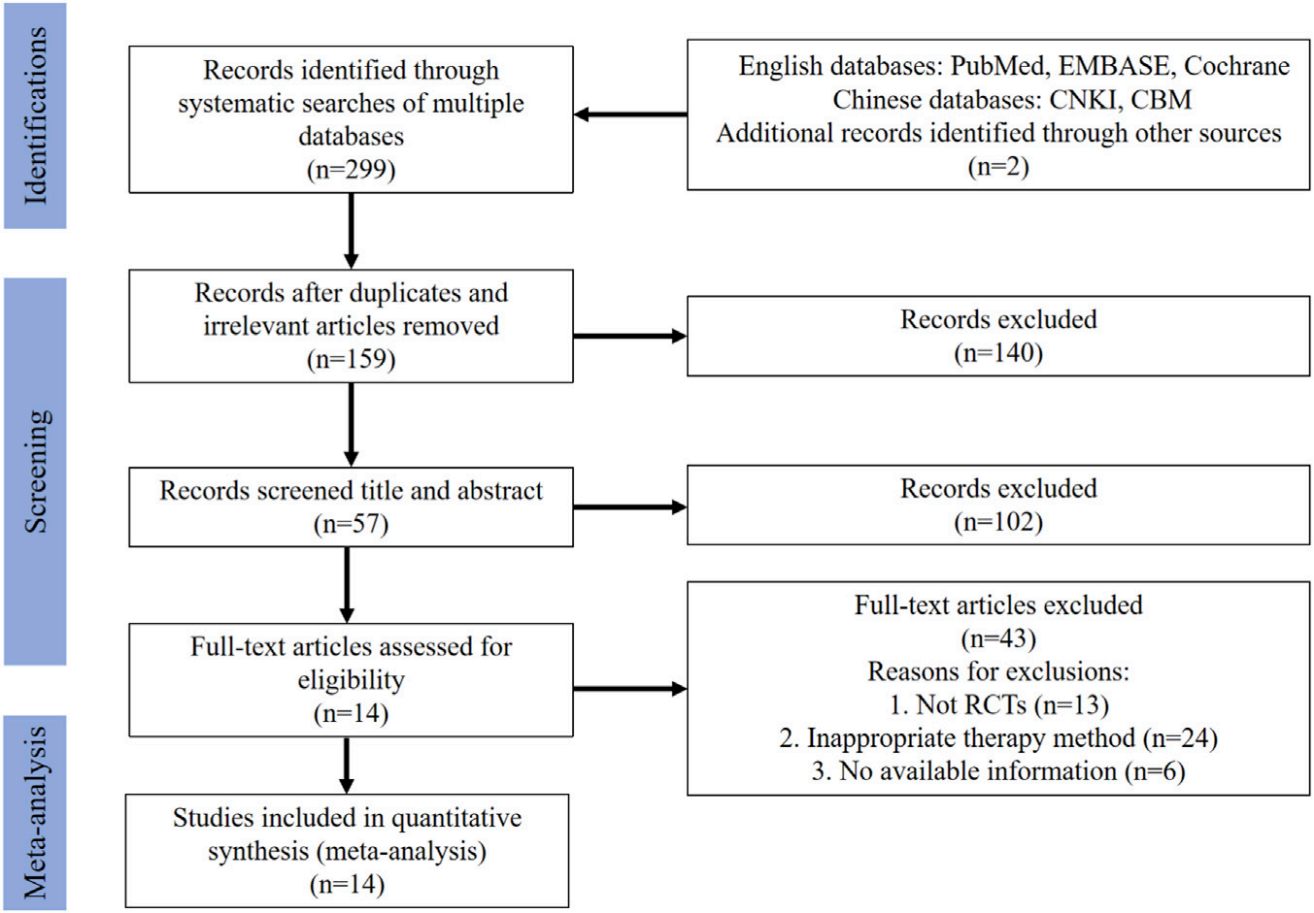
Flow chart of the search and selection process.

We retrieved 299 studies by the initial searches, of which 140 were removed due to duplication and irrelevance. After screening the titles and abstracts, 102 articles were excluded. After screening the full text of the remaining 57 articles, 43 articles were excluded for the following reasons: (1) not RCTs; (2) difference in therapy method between treatment group and control group except for RD; (3) no available information. Ultimately, 14 RCTs involving 1011 participants were eligible for this systematic review (Figure 1) (Huan et al., 2008; Long and Liu, 2009; Mu and Li, 2019; Chen and Chen, 2004; Song, 2008; Yan, 2013; Yu et al., 2010; Sun, 2016; Zheng et al., 2012; Hu and Xu, 2015; Xie, 2017; Gao, 2012; Chen, 2007; He et al., 2021).

### 3.2 Characteristics of included studies

The overall characteristics of 14 qualified studies in this meta-analysis are shown in Table 1. These studies were published between 2004 and 2021. A total of 1011 subjects (59% men), of which 513 participants were randomized to the treatment group and 508 subjects to the control group. The patients were between 30 and 91 years old. The Wagner grade of DFU was mentioned in eight studies (Huan et al., 2008; Long and Liu, 2009; Chen and Chen, 2004; Song, 2008; Yu et al., 2010; Zheng et al., 2012; Hu and Xu, 2015; Gao, 2012), and not mentioned in six studies (Mu and Li, 2019; Yan, 2013; Sun, 2016; Xie, 2017; Chen, 2007; He et al., 2021). The administration of RD was topical application in 12 studies (Huan et al., 2008; Long and Liu, 2009; Mu and Li, 2019; Chen and Chen, 2004; Song, 2008; Yan, 2013; Yu et al., 2010; Sun, 2016; Zheng et al., 2012; Hu and Xu, 2015; Gao, 2012; He et al., 2021) and taken orally in two studies (Xie, 2017; Chen, 2007).

**TABLE 1:** Characteristics of the included RCTs.

Study	Age	Gender (male/Female)	Number of patients (T/C)	Treatment group	Control group	Duration (day)
Huan et al. (2008)	T: 64.07 ± 5.44 C: 64.87 ± 5.91	T: 19/17 C: 18/15	36/33	RD + insulin + antibiotics	Insulin + antibiotics	14
Long and Liu (2009)	54.2 ± 12.5	38/24	32/30	RD + potassium permanganate	Potassium permanganate	40
Mu and Li (2019)	T: 74.9 ± 1.7 C: 74.5 ± 1.8	T: 25/21 C: 26/20	46/46	RD + insulin + antibiotics	Insulin + antibiotics	20
Chen and Chen (2004)	58.3 ± 11.5	40/23	33/30	RD + insulin + antibiotics	Insulin + antibiotics	40
Song (2008)	T: 46–76 C: 42–79	T: 18/15 C: 15/11	33/26	RD + insulin	Insulin	20–40
Yan (2013)	64.07 ± 5.44	36/32	34/34	RD + insulin + hydrogen peroxide	Insulin + hydrogen peroxide	16–20
Yu et al. (2010)	T: 60.21 ± 6.92 C: 60.07 ± 7.01	T: 16/9 C: 14/10	25/24	RD + insulin + antibiotics + anisodamine	Insulin + antibiotics + anisodamine	42
Sun (2016)	62.51 ± 4.08	22/20	12/30	RD + normal saline	Normal saline	20
Zheng et al. (2012)	T: 63.02 ± 4.28 C: 61.68 ± 5.12	T: 29/21 C: 23/26	50/49	RD + insulin + antibiotics + sulfadiazine zinc	Insulin + antibiotics + sulfadiazine zinc	40
Hu and Xu (2015)	62.51 ± 4.08	71/42	59/54	RD + insulin MEBO	Insulin + MEBO	28
Xie (2017)	T: 48.15 ± 4.24 C: 48.69 ± 4.83	T: 19/16 C: 20/15	35/35	RD + lipoic acid + alprostadil	Lipoic acid + alprostadil	14–28
Gao (2012)	40–74	33/19	26/26	RD + insulin + gentamicin + anisodamine	Insulin + gentamicin + anisodamine	42–56
Chen (2007)	T: 60.10 ± 8.18 C: 62.45 ± 7.61	T: 34/28 C: 24/26	62/50	RD + insulin + hypotensor	Insulin + hypotensor	40
He et al. (2021)	37–91	38/23	30/31	Resina Draconis	Alginate	28

DFU: diabetic foot ulcer; T: treatment group; C: Control group; NA: not available; MEBO, moist exposed burn ointment; RD: Resina Draconis.

The characterization of the investigated Resina Draconis preparations was generally insufficient. Most included RCTs described the intervention only as “Resina Draconis” or “Longxuejie” and reported the dosage form and route of administration. Although published phytochemical studies on Dracaena-derived RD have reported major classes of metabolites such as flavonoids, phenolics, and steroids, the clinical trials rarely provided species-level identification, specific botanical drug, authentication procedures, processing/manufacturing details, or phytochemical standardization of the investigated products. Therefore, the exact composition of the preparations used in most included studies could not be determined from the trial reports. These details have now been summarized in Supplementary Table S3, with unavailable items explicitly marked as “not reported”.

### 3.3 Risk of bias assessment

The methodological quality of the included studies was variable. Although several trials reported randomization, the methods used were often insufficiently described. Reporting of allocation concealment and blinding was incomplete in many studies, and some trials provided only limited information on adverse-event monitoring or selective outcome reporting. Therefore, several domains were judged as having unclear risk of bias, and a small number of studies were judged at high risk in specific domains.

**FIGURE 2:**
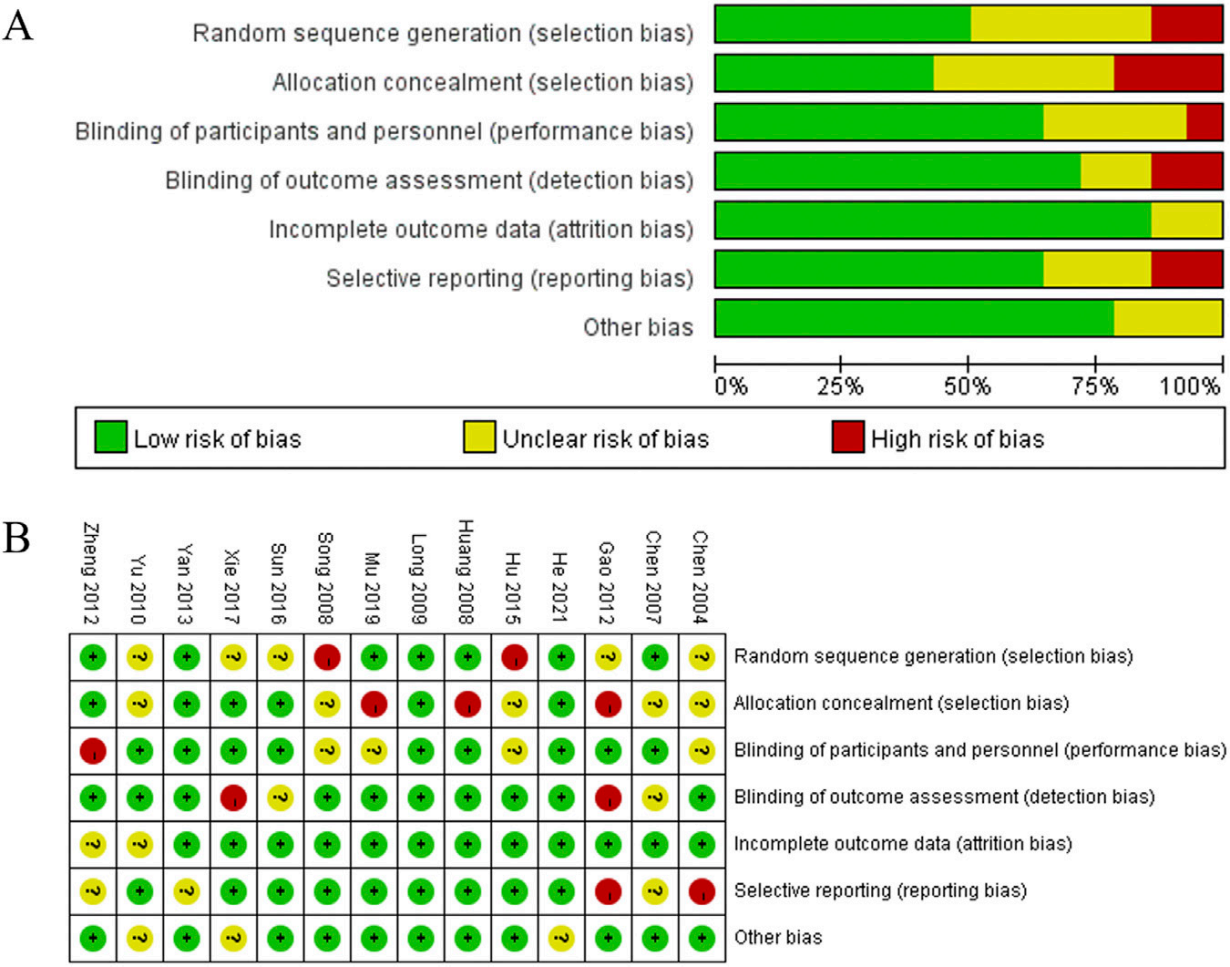
Bias risk assessment figure. **(A)** Percentage diagram of each bias risk evaluation index. **(B)** Bias risk assessment diagram of the included literature.

Overall, the evidence base should be interpreted cautiously because unclear reporting does not provide assurance that bias was absent. The bias risk map is shown in Figure 2. The methodological evaluation of the included RCTs is shown in Table 2.

**TABLE 2:** Methodological evaluation of included RCTs.

Study	Randomization	Concealment	Blinding	Quality of evidence
Huan et al. (2008)	Yes	The allocations were masked unclearly	Yes, single-blind (subjects)	⊕⊕⊕○/Moderate
Long and Liu (2009)	Yes	Yes, allocations were masked	Yes, single-blind (subjects)	⊕⊕⊕⊕/High
Mu and Li (2019)	Yes	No, allocations were not masked	Yes, single-blind (subjects)	⊕⊕○○/Moderate
Chen and Chen (2004)	Yes, random number hiding method	The allocations were masked unclearly	Yes, single-blind (subjects)	⊕⊕○○/Moderate
Song (2008)	Yes	The allocations were masked unclearly	Yes, single-blind (subjects)	⊕⊕○○/Moderate
Yan (2013)	Yes	Yes, allocations were masked	Yes, single-blind (subjects)	⊕⊕⊕⊕/High
Yu et al. (2010)	Yes	The allocations were masked unclearly	Yes, single-blind (subjects)	⊕⊕⊕○/Moderate
Sun (2016)	Yes	Yes, allocations were masked	Yes, single-blind (subjects)	⊕⊕⊕○/Moderate
Zheng et al. (2012)	Yes	Yes, allocations were masked	Yes, double-blinded	⊕⊕○○/Moderate
Hu and Xu (2015)	Yes	The allocations were masked unclearly	No described	⊕⊕○○/Moderate
Xie (2017)	Yes	Yes, allocations were masked	No described	⊕⊕○○/Moderate
Gao (2012)	Yes	No, allocations were not masked	Yes, single-blind (subjects)	⊕○○○/Low
Chen (2007)	Yes	The allocations were masked unclearly	No described	⊕⊕○○/Moderate
He et al. (2021)	No describe	Yes, allocations were masked	No described	⊕⊕○○/Moderate

Methodological evaluation score/grade: ⊕○○○/Low, ⊕⊕○○/Moderate, ⊕⊕⊕○/Moderate, ⊕⊕⊕⊕/High. Note: The higher the score, the more credibl.

### 3.4 Primary outcome: total effective rate of wound healing

**FIGURE 3:**
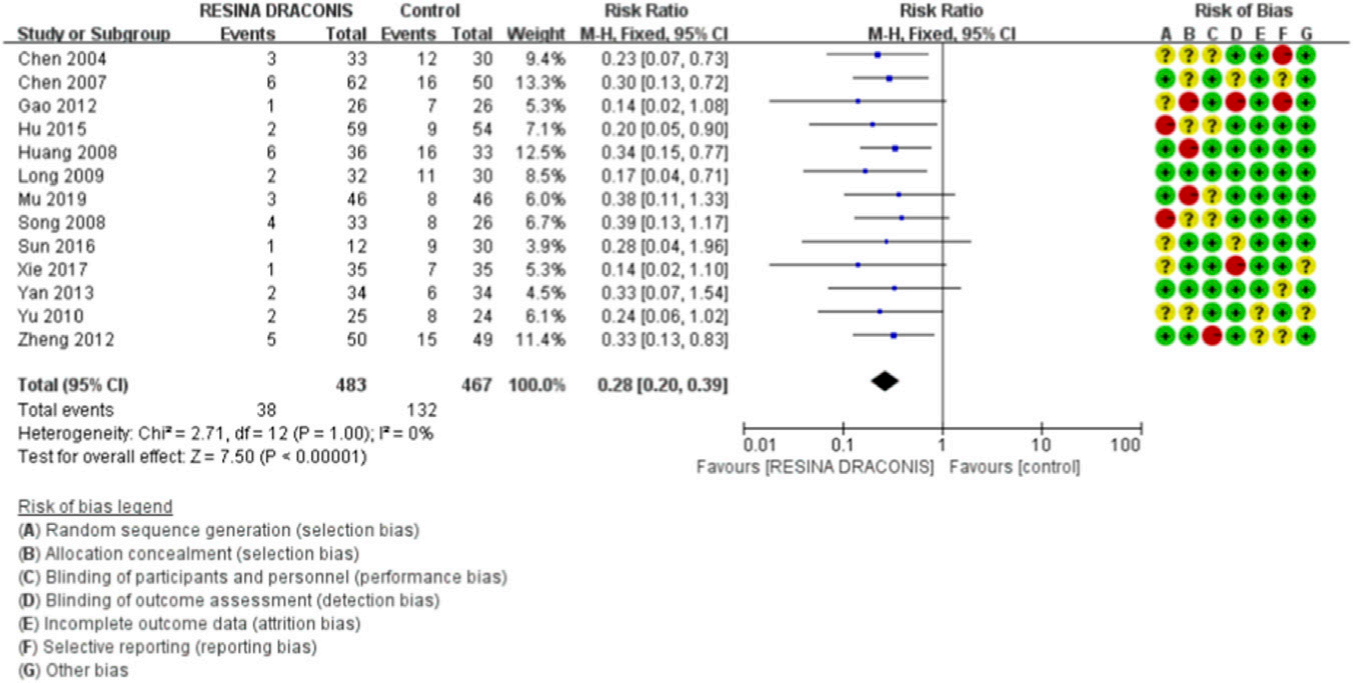
The forest plot showed the effective rate of RD in the treatment of DFU compared with the control group. Note: The meaning of the letter code in the Risks of bias section has been described in section 2.4.

This result was reported in 13 of the 14 RCTs we included (Huan et al., 2008; Long and Liu, 2009; Mu and Li, 2019; Chen and Chen, 2004; Song, 2008; Yan, 2013; Yu et al., 2010; Sun, 2016; Zheng et al., 2012; Hu and Xu, 2015; Xie, 2017; Gao, 2012; Chen, 2007). A total of 950 subjects (483 in RD group and 467 in control group) were included in this analysis. The treatment inefficiency of the RD group was 7.9%, and the treatment inefficiency of the control group was 28.2%. Meta-analysis shows that RD can alleviate the development of DFU to a certain extent and play a therapeutic role (RR = 0.28; 95%CI = 0.20–0.39; *P* < 0.00001). The heterogeneity of the main outcome indicators is extremely low, indicating that the results should be interpreted cautiously because of methodological limitations (Heterogeneity: *P* = 1.00; I^2^ = 0%) (Figure 3).

It can be seen from the bias risk map of Figure 3 that the partial bias risk of some of the included studies is uncertain or has a potential high bias risk. However, due to the extremely low overall heterogeneity of the study, and we used RE for analysis, the results did not change, so these findings should be interpreted with caution because several studies had unclear or high risk of bias.

### 3.5 Secondary outcomes

We also counted C-reactive protein (CRP) and adverse events as secondary outcomes. Meta-analysis of three studies (Mu and Li, 2019; Yan, 2013; Sun, 2016) demonstrated that RD had significant effect on reducing CRP in patients with DFU, compared with control (MD: −1.99, 95%CI: −2.99 to −0.99, *P* < 0.0001; I^2^ = 4%). Adverse events were mentioned in four studies (Song, 2008; Sun, 2016; Zheng et al., 2012; Hu and Xu, 2015), all of which indicated that no obvious adverse events occurred in any of patients. The remaining studies did not mention adverse events during the use of RD.

### 3.6 Non-nested analysis: healing time

Since only topical studies reported the wound-healing time. The oral-administration RCTs did not provide healing time. Therefore, a subgroup analysis for this outcome cannot performed. We performed healing-time synthesis in two analyses: (1) a primary analysis including all studies with extractable healing-time data, and (2) a parallel exploratory analysis restricted to studies that reported Wagner-grade stratified healing outcomes. These are not nested analyses; the Wagner-based analysis is an exploratory comparison within the subset of studies with available grade-stratified reporting. The two pooled effects are not directly comparable as nested estimates because the analysis sets differ in study composition and reporting basis.

**FIGURE 4:**
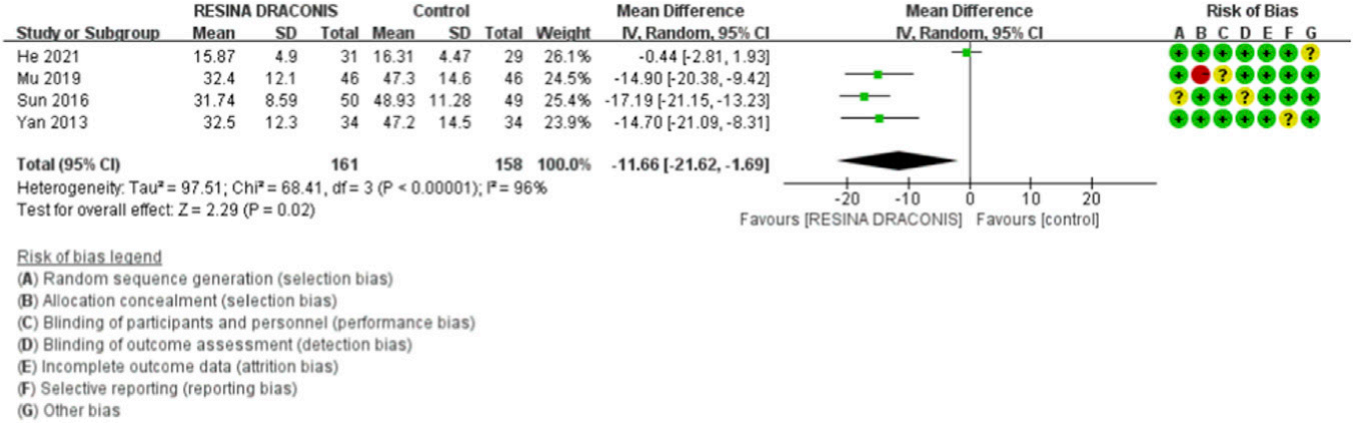
The forest plot showed the healing time of RD in the treatment of DFU compared with the control group.

Ten studies (Long and Liu, 2009; Mu and Li, 2019; Chen and Chen, 2004; Song, 2008; Yan, 2013; Yu et al., 2010; Sun, 2016; Zheng et al., 2012; Hu and Xu, 2015; He et al., 2021) reported wound healing times. One study (Hu and Xu, 2015) could not be included in the pooled analysis due to differing classification methods, while two others (Song, 2008; Yu et al., 2010) were excluded due to incomplete data. Four studies (Long and Liu, 2009; Chen and Chen, 2004; Zheng et al., 2012; He et al., 2021) reported healing times for diabetic foot ulcers at different stages, while the remaining three studies (Mu and Li, 2019; Yan, 2013; Sun, 2016) reported wound healing times for all patients within the same cohort. The meta-analysis of total healing time demonstrated that RD significantly shortened healing time in diabetic foot ulcer patients compared to the control group (MD = −11.66, 95%CI = −21.62 to −1.69; *P* ≤ 0.02; I^2^ = 96%; (Figure 4). To explore whether wound severity may influence healing kinetics, a separate parallel exploratory analysis was performed using the subset of RCTs that reported outcomes stratified by Wagner grade. This analysis is exploratory and should not be interpreted as a nested subgroup of the primary healing-time synthesis.

**FIGURE 5:**
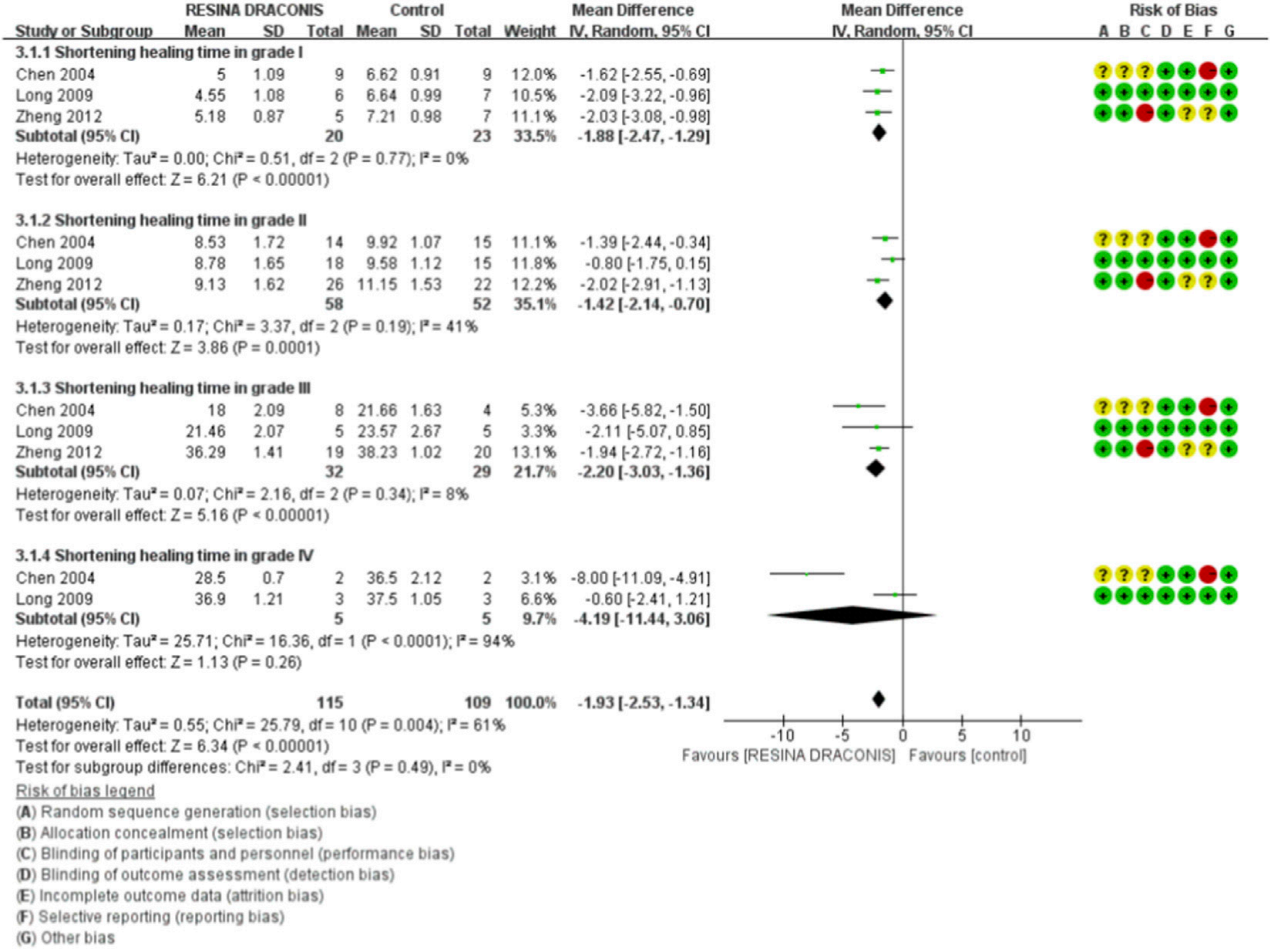
Subgroup analysis showed the length of healing time of RD treatment for different grades of DFU.

To further explore the impact of wound severity on healing kinetics, a separate analysis was performed on the subset of RCTs that provided healing data stratified by Wagner grades. Figure 5 shows the subgroup analysis results of RD reducing the healing time of different grades of wounds. The meta-analysis results for RD reducing wound healing time across different grades are as follows: Grade I (Long and Liu, 2009; Chen and Chen, 2004; Zheng et al., 2012) (n = 43, MD: −1.87, 95%CI: −2.46 to −1.87, *P* < 0.00001, I^2^ = 0%), Grade II (Long and Liu, 2009; Chen and Chen, 2004; Zheng et al., 2012) (n = 110, MD: −1.43, 95%CI: −1.99 to −0.88, *P* < 0.00001, I^2^ = 41%), and Grade III (Long and Liu, 2009; Chen and Chen, 2004; Zheng et al., 2012) (n = 61, MD: −2.14, 95%CI: −2.84 to −1.43, *P* < 0.00001, I^2^ = 8%) demonstrated significant efficacy in patients with diabetic foot ulcers. However, RD showed no statistically significant reduction in wound healing time for patients with grade IV (Long and Liu, 2009; Chen and Chen, 2004) diabetic foot ulcers (*P* = 0.26). After the combined analysis of the above outcome indicators, we found that compared with the control group, the RD group had a significant advantage in the cure time of each grade of DFU (MD = −1.93, 95%CI = −2.53 to −1.34; P < 0.00001) (Figure 5). Obviously, RD group is better than the control group in this index. Detailed baseline/contextual characteristics and data-inclusion mapping are presented in Supplementary Table S4.

### 3.7 Sensitivity analysis

It is clear that the heterogeneity of the outcome of healing time is very high, so we conducted a sensitivity analysis of this outcome. We excluded He and her colleague’s study (He et al., 2021) and found that heterogeneity decreased significantly (From I^2^ = 96% to I^2^ = 0%). This shows that their study is the main reason for the excessive heterogeneity of the outcome. However, after excluding this study, we found that the results did not change significantly. Therefore, we believe that although this study has caused too much heterogeneity, it does not affect the conclusion in essence, so it is not necessary to exclude this study.

**FIGURE 6:**
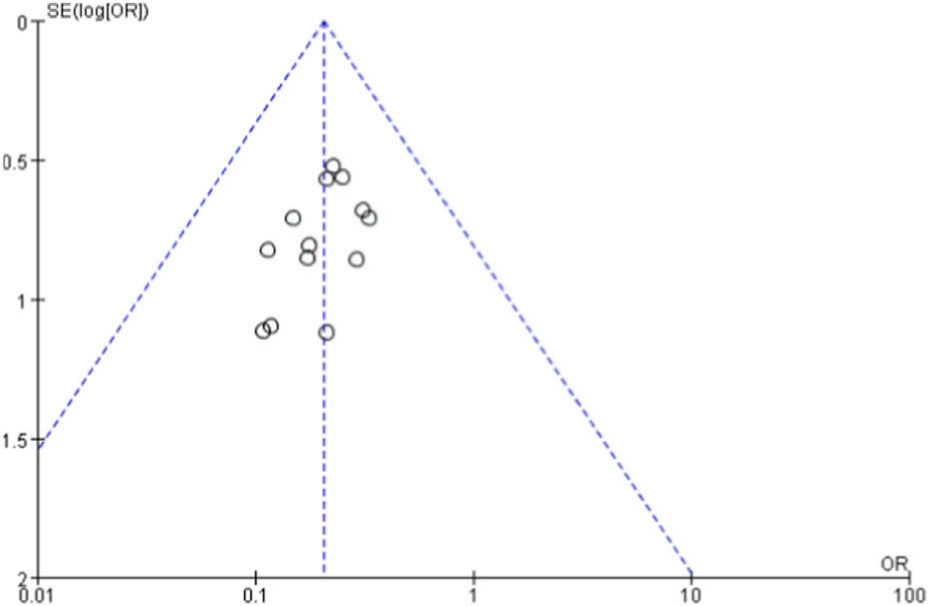
Funnel plot to assess the potential publication bias in the main outcome.

For the rigor of the study, we evaluated the publication bias of the main outcome. As shown in Figure 6, the distribution of the two ends of the funnel plot of 14 studies (Huan et al., 2008; Long and Liu, 2009; Mu and Li, 2019; Chen and Chen, 2004; Song, 2008; Yan, 2013; Yu et al., 2010; Sun, 2016; Zheng et al., 2012; Hu and Xu, 2015; Xie, 2017; Gao, 2012; Chen, 2007; He et al., 2021) is relatively symmetrical, which indicates that there may be less publication bias.

## 4 Discussion

As a serious complication of DM, the adverse outcome of DFU has become an important factor in determining the quality of life and economic burden of patients. RD, as a traditional Chinese herbal medicine, has important effects such as antibacterial, anti-inflammatory and promoting wound healing. It is widely used in the treatment of various ulcers in clinic. RD has been used in the treatment of DFU in China, but its efficacy is not clear. In this study, a systematic review of this reliable evidence method was used to clarify the therapeutic effect of RD on patients with DFU. The results of Meta-analysis showed that compared with the control group, preparations reported as RD were associated with improved clinical outcomes., but also significantly shorten the wound healing time of grade I-III DFU. In addition, RD may play an anti-inflammatory role by reducing the body’s CRP level and indirectly promote the healing of DFU. In terms of drug safety, no obvious adverse reactions have been found.

Previous animal studies have suggested that some metabolites in RD-related preparations may regulate the oxidative stress-inflammatory axis like AGE-RAGE and downstream inflammatory signals, associated with diabetic chronic wounds, thus supporting the clinically observed reduction of CRP and promoting the regeneration of diabetic wound skin tissue. Among them, loureirin B was confirmed to be one of the active metabolites in this model (Jin et al., 2025). This is biologically consistent with our clinical findings. The available evidence suggests a possible association between RD-labelled preparations and improved wound-healing outcomes. However, it should be emphasized that the RCTs included in this study generally lack a unified component characterization and mechanism biomarker (except CRP) report, so the effect cannot be directly extrapolated to a single component such as dracorhodin in all preparations, and the related discussions are only supportive mechanism speculation (Sun et al., 2021; Wang et al., 2025; Sun et al., 2019). Our study indicated that preparations reported as RD in the included trials were associated with improved DFU wound-healing outcomes compared with control therapy. Wound healing time, as an important evaluation index of wound healing, largely determines the disease burden of patients. Previous studies have suggested that RD helps skin wounds heal faster and more firmly by promoting cell movement, enhancing tissue repair, and stimulating vascular growth (Pona et al., 2019). This theory explains our results to some extent. Although the index of wound healing time showed high heterogeneity, we attributed it to the study (He et al., 2021) of He and her colleagues according to sensitivity analysis, and found that it did not affect the conclusion in essence. The results of Meta-analysis showed that RD had statistical significance in shortening the wound healing time of DFU patients. The results of subgroup analysis showed that the healing time of DFU in grade I, grade II and grade III was significantly shortened after the application of RD. It can be found that the lower the level, the shorter the healing time of DFU, the better the therapeutic effect of RD on DFU, and the greater the benefit of patients. Although the results showed that there was no significant difference in the distribution of grade IV DFU treated with RD, which was different from our expectations, it could also be explained by the characteristics of grade IV DFU, namely, ischemia and gangrene. When DFU progresses to local ischemia and gangrene, the blood supply and nutrition of the wound are basically interrupted, so it is difficult for RD to play its therapeutic role. In addition, too small sample size (n = 10) may also lead to the study failed to detect statistical significance. In the future, further large-scale clinical trials are needed to obtain a reliable conclusion of RD for more severe DFU.

Infection, as an important mechanism for inducing DFU, often triggers the body‘s inflammatory response, thereby delaying wound healing. CRP is recognized as a sensitive biological marker of inflammation. When inflammation occurs in the body, it drives a large amount of liver synthesis through the IL-6/STAT3 signaling pathway, and CRP is transformed into mCRP in an acidic environment to activate the complement system and pro-inflammatory pathway, thereby amplifying the local inflammatory response and causing a series of pathological changes in the body (Rizo-Téllez et al., 2023; Zhou et al., 2024). In this systematic review, the level of CRP was significantly decreased after treatment with RD. This may be related to a broader modulation of oxidative stress-inflammation pathways reported for RD metabolites (Sun et al., 2019; Liu et al., 2021), which is consistent with the observed CRP reduction (Rizo-Téllez et al., 2023; Zhou et al., 2024). In short, preparations reported as RD may indirectly promote DFU healing through anti-inflammatory and pro-repair biological effects; however, this mechanistic explanation remains inferential because most RCTs lacked detailed composition reporting and did not evaluate mechanistic biomarkers beyond CRP. Therefore, the role of dracorhodin should be interpreted cautiously in this context and cannot be assumed for all included preparations.

The potential adverse reactions of the drug will limit its clinical application. In terms of safety, the evidence base is limited by incomplete reporting. Among the 14 included RCTs, only four studies (Song, 2008; Sun, 2016; Zheng et al., 2012; Hu and Xu, 2015) explicitly stated that no obvious adverse events occurred during the study period, whereas the remaining studies did not report adverse events. Therefore, the current findings should not be interpreted as definitive evidence of safety. Chen and his colleagues found that RD and its crude extract were non-cytotoxic (Chen et al., 1994). This is consistent with the previous conclusion that there is no significant toxicity of RD obtained by Fan and her colleagues on the toxicological study of RD (Fan et al., 2014). From a pharmacological and clinical perspective, potential adverse reactions may occur under certain conditions. For topical use on DFU wounds, possible events include local irritation, pain/burning sensation, contact dermatitis or allergic reactions, especially when the wound area is large/deep, the skin barrier is severely compromised, occlusive dressings are used, or product quality control is inadequate. Because DFU wounds are prone to infection, inappropriate local management or contaminated products could theoretically aggravate local inflammation or infection, although this was not systematically assessed in the included trials. For oral administration, systemic adverse effects and botanical drug-drug interactions are more relevant because DFU patients commonly use antidiabetic agents, antibiotics, antiplatelet and anticoagulant drugs. Although no interaction events were reported, such risks cannot be excluded due to the lack of structured monitoring and reporting. Future RCTs should predefine safety outcomes and actively monitor adverse events and potential interactions.

Compared with previous similar study (Feng et al., 2023) by Feng and his colleagues, our study included more literature and had stronger evidence. In the subgroup analysis of healing time, we specifically analyzed the therapeutic effect of RD on grade IV DFU, and further studied its efficacy. However, our study also has some limitations. A major limitation identified in this review is the insufficient pharmacognostic characterization of the investigated preparations. Most included RCTs did not report source species, authentication procedures, manufacturing details, processing methods, or phytochemical quality-control parameters. Consequently, it is impossible to determine whether the investigated products represented the same botanical drug or comparable preparations. This limitation substantially reduces the interpretability, reproducibility, and generalizability of the available evidence. Secondly, the funnel plot of the included studies showed a certain degree of asymmetry, suggesting that there may be publication bias, which may be related to the fact that RD is a traditional Chinese medicine and only relevant RCTs are currently carried out in China. In the future, it is expected that more RCTs will be carried out internationally to further draw reliable conclusions. What’s more, the scale of RCTs designed by these studies is not large, and potential publication bias may have a greater impact on our conclusions. However, meta-analysis is limited by methodology and has limited ability to solve publication bias. Therefore, we should be more cautious about the results. In the RCTs we included in the study, the use of RD involves oral and external application. However, Given the current reporting structure, route-based heterogeneity cannot be explored for healing time. The apparent discrepancy between the pooled estimate of healing time and the Wagner-stratified exploratory analysis should be interpreted cautiously. These do not represent a single, nested comparison; they are based on different reporting bodies of trials. Therefore, the larger effect in the overall estimate likely reflects heterogeneity in study context and reporting structure (e.g., ulcer-related inclusion criteria, co-treatments, outcome definition, follow-up timing) rather than a direct, isolated grade effect. Future trials should use standardized reporting of Wagner grade, ulcer duration, dose regimen, and healing endpoint definition. There were some problems in the methodological quality of the selected studies. Specific performance in the use of blind law norms. This bias may cause the measurement results to exaggerate the therapeutic effect of RD. It is expected that more high-quality RCTs will be carried out in the future to provide more reliable evidence for the treatment of DFU with RD.

## 5 Conclusion

This systematic review identified potentially favorable clinical outcomes associated with preparations labelled as Resina Draconis in patients with diabetic foot ulcers. However, because most included studies did not adequately define or authenticate the source material and provided limited information regarding processing and quality control, the current evidence remains insufficient to support definitive conclusions regarding the efficacy or safety of a clearly defined Resina Draconis preparation. Future well-designed trials using taxonomically validated and pharmacognostically standardized materials are required.

## Data Availability

The original contributions presented in the study are included in the article/Supplementary Material, further inquiries can be directed to the corresponding author.
