# Supplementary material for: Efficacy and safety of resina draconis as an adjuvant therapy for diabetic foot ulcer: a meta-analysis of randomized controlled trials
Source: Front Pharmacol. 2026 Jul 22;17:1823436. doi: 10.3389/fphar.2026.1823436 (PMC13437936; doi:10.3389/fphar.2026.1823436)
Supplement: Supplementary file 3 [file Table3.docx]

**Supplementary Table S3. The source and details of herbs in RCT**

| **Study** | **Intervention name** | **Dosage form** | **Manufacturer** | **Authentication** | **Manufacturing details** |
| --- | --- | --- | --- | --- | --- |
| Huang 2008^[17]^  DFU: diabetic foot ulcer; T: treatment group; C: Control group; NA: not available; MEBO, moist exposed burn ointment; RD: Resina Draconis. | Resina Draconis | Capsule content wet compress | Mingsheng Pharmaceutical Factory | NMPAC | Not Report |
| Long 2009^[18]^ | Resina Draconis | Capsule content wet compress | Not Report | Not Report | 0.6g Resina Draconis mix with 10mL 75% ethanol |
| Mu 2019^[19]^ | Resina Draconis | Capsule content wet compress | Kam Long Pharmaceutical Co., Ltd. | NMPAC | Resina Draconis mix with iodophor |
| Chen 2004^[20]^ | Resina Draconis | Capsule content wet compress | Gejiu City Pharmaceutical Co., Ltd. | NMPAC | Not Report |
| Song 2008^[21]^ | Resina Draconis | Capsule content wet compress | Guilin Sanjin Pharmaceutical Co., Ltd. | NMPAC | Not Report |
| Yan 2013^[22]^ | Resina Draconis | Capsule content wet compress | Yunnan Yunhe Pharmaceutical Co., Ltd. | NMPAC | Resina Draconis mix with iodophor |
| Yu 2010^[23]^ | Resina Draconis | Capsule content wet compress | Yunnan Yunhe Pharmaceutical Co., Ltd. | NMPAC | Resina Draconis mix with normal saline |
| Sun 2016^[24]^ | Resina Draconis | Capsule content wet compress | Not Report | Not Report | Resina Draconis mix with iodophor |
| Zheng 2012^[25]^ | Resina Draconis | Oral capsule | Yunnan Yunhe Pharmaceutical Co., Ltd. | NMPAC | Not Report |
| Hu 2015^[26]^ | Resina Draconis | Capsule content wet compress | Peking Tongrentang Pharmaceutical Factory | Not Report | 0.4g Resina Draconis mix with 5mL 75% ethanol |
| Xie 2017^[27]^ | Resina Draconis | Oral capsule | Yunnan Yunhe Pharmaceutical Co., Ltd. | NMPAC | Not Report |
| Gao 2012^[28]^ | Resina Draconis | Capsule content wet compress | Zhaoqing Xinghu Pharmaceutical Co., Ltd. | NMPAC | Not Report |
| Chen 2007^[29]^ | Resina Draconis | Oral capsule | Nanning Hengying Pharmaceutical Co., Ltd. | NMPAC | Not Report |
| He 2021^[30]^ | Resina Draconis | Capsule content wet compress | Not Report | NMPAC | Not Report |

NMPAC: National Medical Products Administration of China
