# Supplementary material for: Efficacy and safety of resina draconis as an adjuvant therapy for diabetic foot ulcer: a meta-analysis of randomized controlled trials
Source: Front Pharmacol. 2026 Jul 22;17:1823436. doi: 10.3389/fphar.2026.1823436 (PMC13437936; doi:10.3389/fphar.2026.1823436)
Supplement: Supplementary file 4 [file Table4.docx]

**Supplementary Table S4. Study-level data used in healing-time analyses**

| **Study** | **Intervention formulation** | **Number of Patients (T/C)** | **Resina Draconis** | | **Control** | | **Wagner grade data** | | | | | |
| --- | --- | --- | --- | --- | --- | --- | --- | --- | --- | --- | --- | --- |
|  |  |  | Mean | SD | Mean | SD | Grade | Number of Patients (T/C) | Resina Draconis | | Control | |
|  |  |  |  |  |  |  |  |  | Mean | SD | Mean | SD |
| Long 2009^[18]^ | Topical application | 32/30 | Not Report | | | | I | 6/7 | 4.55 | 1.08 | 6.64 | 0.99 |
|  |  |  | Not Report | | | | II | 18/15 | 8.78 | 1.65 | 9.58 | 1.12 |
|  |  |  | Not Report | | | | III | 5/5 | 21.46 | 2.07 | 23.57 | 2.67 |
|  |  |  | Not Report | | | | IV | 3/3 | 36.9 | 1.21 | 37.5 | 1.05 |
| Zheng 2012^[25]^ | Topical application | 50/49 | Not Report | | | | I | 5/7 | 5.18 | 0.87 | 7.21 | 0.98 |
|  |  |  | Not Report | | | | II | 26/22 | 9.13 | 1.62 | 11.15 | 1.53 |
|  |  |  | Not Report | | | | III | 19/20 | 36.29 | 1.41 | 38.23 | 1.02 |
|  |  |  | Not Report | | | | IV | N/A | N/A | N/A | N/A | N/A |
| Chen 2004^[20]^ | Topical application | 33/30 | Not Report | | | | I | 9/9 | 5.0 | 1.09 | 6.62 | 0.91 |
|  |  |  | Not Report | | | | II | 14/15 | 8.53 | 1.72 | 9.92 | 1.07 |
|  |  |  | Not Report | | | | III | 8/4 | 18.0 | 2.09 | 21.66 | 1.63 |
|  |  |  | Not Report | | | | IV | 2/2 | 28.5 | 0.7 | 36.5 | 2.12 |
| Yan 2013^[22]^ | Topical application | 34/34 | 32.5 | 12.3 | 47.2 | 14.5 | Not Report | | | | | |
| Sun 2016^[24]^ | Topical application | 12/30 | 31.74 | 8.59 | 48.93 | 11.28 | Not Report | | | | | |
| Mu 2019^[19]^ | Topical application | 46/46 | 32.4 | 12.1 | 47.3 | 14.6 | Not Report | | | | | |
| He 2021^[30]^ | Topical application | 30/31 | 15.87 | 4.9 | 16.31 | 4.47 | Not Report | | | | | |
